# 

**Published:** 1910-07-23

**Authors:** 


### A MEDICAL HOMES DIRECTORY.

The 1910 edition of "Medical Homes for Private Patients" (The Scientific Press, Ltd., 29 Southampton Street, W.C., 6d. net), is an excellent little summary of all the reputable nursing homes which should prove very useful to patients and practitioners alike. The lists of practising specialists and consultants have been added to but are still incomplete, and should be carefully looked over in the next edition, since they contain names of men now, alas! no longer with us.

### APPOINTMENTS.

Hubert Pinto-Leite, B.A. Cantab, M.R.C.S., L.R.C.P., has been appointed Clinical Assistant at the Royal London Ophthalmic Hospital, and re-appointed Clinical Assistant to the Aural Department at St. George's Hospital.

### Gifts and Bequests.

Prince Francis of Teck has received the following in response to his appeal on behalf of the Middlesex Hospital: Lord and Lady Pirrie, £250; Lord Portman, £200; and Messrs. Linton, Clarke and Co., £105.

The Wood Green Hospital Council understands that a legacy of £8,000 has been left by the late Mr. Leggatt, of Hornsey, to be divided between the Wood Green and District Hospital, the Royal Free Hospital, and the Great Northern Hospital.

The late Dr. Florence Nightingale Boyd, M.D., senior surgeon of Charing Cross Hospital, has left £100 to the New Hospital for Women, Euston Road, N.W. (her old hospital), £50 to the Pathological Department of the hospital, and £100 to the London School of Medicine for Women.

The estate of Baron Sir John Henry William Schröder, a baronet of the United Kingdom and a baron of the Kingdom of Prussia, head of the firm of Messrs. J. Henry Schröder and Co., merchant bankers, has been sworn at £2,079,611 gross and at £1,970,927 16s. 2d. net. His bequests include £10,000 to the German Hospital, Dalston; £1,000 each to the Windsor Royal General Dispensary and Princess Christian's Windsor District Nursing Fund.

At the monthly meeting of the Executive Committee of the General Infirmary, Worcester, it was announced that a legacy of £2,000, with £244 9s. 5d. interest, had been received from the trustees of the late Mr. John Corbett, of Droitwich. The Chairman remarked that this would be a great help to the institution's funds, and it was represented by another member of the committee that £500 had been lost by selling out Corporation stock.

### JOTTINGS.

About £48,000 has now been received at the Mansion House as the result of the collections at the churches and chapels of the Metropolis on Hospital Sunday.

The Queen has become Patroness-in-Chief of the Samaritan Free Hospital for Women, and has continued her patronage of the Victoria Home for Invalid Children at Margate.

The committee for the removal of King's College Hospital to South London have received a cheque for £100 from Viscountess Hambleden, this being her fourteenth donation in aid of the fund.

A dropped "s" in our last issue made it appear that Mr. David Beatty had established the "Peter" cot in the ophthalmic wards of the Leicester Infirmary. The gift was from Mrs. David Beatty.

Elgar's *Dream of Gerontius* was given in the hall of Arundel Castle last week by the Queen's Hall Orchestra and Dr. Coward's Sheffield Choral Union. The Duchess of Norfolk, who organised the concert, will give the profits to the Arundel Emergency Hospital and Littlehampton Hospital building fund.

Owing to an alteration of the rules of the Norfolk and Norwich Hospital the Board of Management is now empowered to recommend for election as Vice-Presidents any persons who are held to have rendered exceptional services to the hospital. Hitherto the Bishop of Norwich has been the only holder of the office.

The new Capel wing of the Pontypool and District Hospital, the cost of which has generously been paid by Mr. and Mrs. J. C. Hanbury, of Pontypool Park, in memory of their only son, Master Capel Hanbury, who died rather more than twelve months ago, was opened by Mr. A. A. Williams, J.P., C.C., on behalf of Mr. and Mrs. Hanbury last week. Mr. B. Nicholas, J.P., C.C., chairman of the executive committee of the hospital, presided.

Col. R. H. Rawson, M.P., who presided at the recent annual festival dinner of the Earlswood Asylum, Redhill, stated that owing to defects they had had to spend over £53,000 in entirely rebuilding a great part of the asylum, and £30,000 was still required for further structural alterations. Col. Rawson thought they could deal properly with the feeble-minded only when the authorities had power of detention, and suggested that to meet sentimental objections they might substitute some other name for "asylum." During the evening subscriptions to the amount of £1,692 were announced.

Military hospitals perhaps receive less attention as a rule from Royalty than civil hospitals, but last week, at any rate, this was quite reversed. His Majesty King George and the Queen inspected the Cambridge and Connaught Hospitals at Aldershot, which together possess nearly a thousand beds, and were received by Surgeon-General Sir T. J. Gallwey, Principal Medical Officer to the command, and at the latter by Lieut.-Col. H. M. Sloggett, the officer in charge, and the matron, Miss Cox. Their Majesties at both hospitals showed great interest in the treatment of consumption in the open-air wards.

### TO HOSPITAL SECRETARIES AND OTHERS.

We invite contributions from any of our readers, and shall be glad to pay, according to length, for items of news for the institutional section (including appointments, resignations, gifts, etc.) which we may publish. All payments for manuscripts used are made as early as possible after the beginning of each quarter.

The King has become patron of St. George's Hospital, and has allowed the new children's wing at Yarmouth Hospital to receive the name of King Edward's Ward.

An $x$-ray and electrical treatment department, costing £1,000, has just been presented to the Royal Surrey County Hospital, Guildford, by Miss Helen Crooke, in memory of her father, the late Mr. F. A. Crooke, of Guildford.

London Hospital Medical School.—In the course of his address to the students at the annual prize distribution on Monday last the Hon. Harry Lawson, M.P., said they were delighted that lately they had had the opportunity of seeing a glorious picture of the advance of enlightenment and humanity in the charming book which their ideal Secretary, Mr. Morris, had written, and in the pages of which he had told the history of the London Hospital. He was not sure that the House Committee had so much to pride themselves on. In the old days it seemed that their predecessors were in the habit of sitting from ten to four and of then adjourning "to the Angel and Crown tavern in Whitechapel for the better transaction of the business of the infirmary." Whatever time Mr. Holland—whom they were all glad to see back, and to know that he had been restored to that buoyant and inspiring energy to which they all owed so much—whatever time he might give, and it was practically all his time, his colleagues could not claim to give as much as the good men of the old days. They certainly did not claim some of their prerogatives, because he understood that in past times the House Committee decided which case should go to the surgeons and which to the physicians. They were very far off, he was glad to think, from the time when members of the staff were divided into rival camps who quarrelled over those whom they called the "miserable objects" of their treatment. They were equally far off from those days of terror when the inmates of the hospital sought to escape by night from the surgeon's knife; when a "black list" was kept of runaways, to whom all further treatment was denied; and when the only man who, after committing such an offence, had the chance, not of coming and cutting again, but of coming and being cut again, was one who had lost a leg in the service of his country. Coming nearer home, it was even more satisfactory to think that they had almost forgotten the days when the medical students were divided into gangs, each of which fought for its own chief, not only with words, but with blows, or, even more recently, when, "disguised in liquor," they painted the East-end red and had to be severely handled by the hall porter. It was a good thing that those days had passed. Of all the changes that had come over London and the medical profession there was none more creditable than what was really the transformation of the medical student. The average figure of merit had gone up to an astounding extent. He did not think they could attribute it all to the general softening of manners or to the immense improvement in medical and surgical training, and the excellent schools and their full equipment. Perhaps it was due more than anything else to the higher esteem of the great profession of the art of healing, which opened up to every student such unlimited possibilities in the saving of human life and the alleviation of human pain. The London Hospital Medical College was the first school to be attached to a great hospital, and had maintained its pride of place. He thought he might speak for his colleagues and say the House Committee was proud of the Medical School and of its scholars. The Hon. Sydney Holland moved a vote of thanks to Mr. Lawson. The chair was taken by Mr. William Hoare.

### COMING EVENTS OF THE WEEK.

[Communications for this column must be sent to 29 Southampton Street, Strand, before noon on Wednesday.]

Saturday, July 23.—Royal Sanitary Institute, Sessional Meeting, Torquay.

Annual garden party, Frimley Sanatorium.

Sunday, July 24.—Visiting day at the hospitals.

Monday, July 25.—Summer School, North Wales Temperance Federation, commences Colwyn Bay.

Tuesday, July 26.—B.M.A. Presidential Address, St. James's Hall, 8.30.

Wednesday, July 27.—International Congress on Administrative Science, Brussels.

B.M.A. Reception, Guildhall, 8.30 p.m.

B.M.A. Luncheon and Garden Party, St. Bartholomew's Hospital.

Garden Party, Bethlem Hospital.

Special Medical Service, Westminster Abbey, 3 p.m.

Special Medical Service, Westminster Cathedral, 10.30 a.m.

B.M.A. (Medical Sociology section), Prof. Moore opens discussion on the Economic Basis of Hospital Management, 10 a.m.

Thursday, July 28.—B.M.A. Annual Dinner, Connaught Rooms, 7.30 p.m.; Ladies' Dinner, B.M.A., Hotel Cecil, 7.30.

B.M.A. Garden Party, Ranelagh Club.

Matinée in aid of Middlesex Hospital, Boudoir Theatre, Pembroke Gardens, 3.30.

Friday, July 29.—Reception B.M.A., Natural History Museum, South Kensington, 8.

Saturday, July 30.—Sir Wm. Treloar's Cripples' Home, B.M.A. Members Visit; Excursion to Bath, B.M.A., Paddington, 10.30.

The reappointment of Mr. D. J. Galloway, M.D., to be an unofficial member of the Legislative Council of the Straits Settlements is gazetted.

THE HOSPITAL. July 23, 1910.

*Name* ..................................................

*Address* ..................................................

..................................................

**This Coupon must accompany manuscript or contributions intended for The Hospital.**

